# Supplementary material for: Gaps in kidney stone disease management: From clinical theory to patient reality
Source: Urolithiasis. 2024 Apr 9;52(1):61. doi: 10.1007/s00240-024-01563-6 (PMC11004051; doi:10.1007/s00240-024-01563-6)
Supplement: Supplementary file 1 — Supplementary file1 (DOCX 79 KB) [file 240_2024_1563_MOESM1_ESM.docx]

**Gaps in Kidney Stone Disease Management: From Clinical Theory to Patient Reality.**

Agnieszka Pozdzik M.D., Ph.D ^1, 2*†^, Viridiana Grillo ^3†^, Khashayar Sakhaee M.D,^2^

^1*^Department of Nephrology and Dialysis, Kidney Stone Clinic, University Hospital Brugmann, Place A. Van Gehuchten 4, Brussels 1020, Belgium

^2^ Faculty of Medecine, Université Libre de Bruxelles (ULB), Route de Lennik 808, Brussels 1070, Belgium

^3^ MFP Haute Ecole de Vinci, Institut Paul Lambin, Place d’Alma 3, Brussels, 1200, Belgium

^4^ Department of Internal Medicine, and Charles and Jane Pak Center for Mineral Metabolism and Clinical Research, , University of Texas Southwestern Medical Center, Harry Hines Boulvards 5939, Dallas, 75390, Texas, USA

*Corresponding author. E-mail: [Agnieszka.Pozdzik@chu-brugmann.be](mailto:Agnieszka.Pozdzik@chu-brugmann.be)

^†^These authors contributed equally to this work

**Abstracts:**

With nephrolithiasis projected to affect 25% of the global population in the next three decades, there is an urgent call for innovative management strategies to prevent and reduce stone recurrence. This study aims to explore the evolving management needs in nephrolithiasis from both patient and healthcare provider perspectives. An expert-collaborative online survey comprising 10 targeted questions on kidney stone management was developed and disseminated. This survey was designed to gather comprehensive insights from patients, physicians and dietician and other person in the field of nephrolithiasis. Analysis of responses from 120 participants, including 45 nephrologists, 38 dieticians, 11 urologists, and 14 kidney stones patients followed in our hospital, revealed critical insights. A significant 97.5% emphasized the necessity of optimizing daily water intake, and 94.1% recognized the need for practical dietary modifications. Additionally, 88.3% of respondents found timely hydration reminders beneficial. Notably, monitoring urine color and pH was valued by 85% and 84.3% of the participants, respectively. A striking disparity emerged in the perception of fatigue and wellness monitoring, with 65% of patients prioritizing fatigue monitoring, a view less shared by healthcare professionals. Similarly, 71% of patients deemed wellness monitoring essential, highlighting a gap in understanding between patients and their caregivers. This study underscores the critical need for more tailored guidance on hydration strategies and the promise of remote urine parameters monitoring in nephrolithiasis management. The findings strongly advocate for a patient-centered approach, aligning medical recommendations with patient lifestyles and experiences, to enhance the effectiveness of prevention.

**Key words:**

Kidney stones disease

Kidney stone management

Kidney stone prevention

Fluid intake

Urine monitoring

**INTRODUCTION**

Kidney stone disease (KSD), is rapidly emerging as a significant public health concern, marked not only by its excruciating pain but also by complex systemic implications (1). Traditionally perceived as an acute condition, KSD is now recognized as a chronic disorder of mineral metabolism (2). This paradigm shift is pivotal, given the disease's rising prevalence and substantial impact on patients' quality of life (3, 4).

The epidemiology of KSD is exhibiting an alarming trend (5). Over the past four decades, both the incidence and prevalence of kidney stones have escalated. This disease, once an incidental asymptomatic finding, is now often a painful recurrent disorder with considerable morbidity. The reported recurrence rate of KSD varies significantly, ranging from 6.1% to 66.9%, depending on various factors. These figures underscore the disease's chronic nature and the challenges in managing its recurrence. Moreover, risk factors for symptomatic kidney stone recurrence include younger age, male gender, family history of stones, obesity, and pregnancy, among others (6).

In the United States, the economic burden of KSD has been staggering. From $2.1 billion spent on stone disease in 2000, the projected expenditure is set to reach $4.1 billion by 2030 (7, 8). These figures represent only the direct costs, such as surgical therapy, which is the primary driver of these expenses. When considering indirect costs, including missed workdays, the economic impact becomes even more significant. For instance, the total indirect cost of stone disease in the United States, based on a privately insured population, was $775 million, accounting for an average of 3.1 million missed workdays per year due to stone disease (9).

Despite considerable advances in the pathophysiological understanding and development of prevention guidelines, there remains a notable gap in managing this growing epidemic. Current prevention strategies, includes increased fluid intake, dietary modifications, and medication (10). Regardless of significant improvements in KSD management, patient adherence to preventive pharmacological therapy is low (42% for alkali citrate and 52% for hydrochlorothiazide) (11). Less than one-third (30.2%) of the 7980 adults with kidney stones who were prescribed therapy adhered to their regimen. It noted that adherence rates differed depending on the type of treatment prescribed: 42.5% for thiazides, 40.0% for allopurinol, and only 13.4% for citrate therapy (12). The main reason for non- adherence in the alkali citrate group was the high number of pills required, while adverse drug effects were the primary concern in the hydrochlorothiazide group. Younger patients showed poorer adherence compared to older patients with chronic conditions and multiple medications (11).

Slightly more than half (51.1%) of the 8,950 patients who met the study eligibility criteria were adherent to preventive pharmacological therapy. The frequency of emergency room visits, hospitalizations and stone-related surgery was significantly lower in adherents than in non-adherents (13).

These findings highlight the issue of low adherence to medication regimens among patients with kidney stones and suggest the need for improved strategies to increase patient adherence . This highlights the urgent need for more effective strategies to enhance medication adherence among KSD patients. This gap between clinical recommendations and patient adherence contributes to the high recurrence rate of kidney stones, which can be as high as 50% within five years of an initial episode in adults and 3 years in children (14).

Recognizing these challenges, the International Society of Nephrology has called for a transformative change in managing kidney disease, focusing more on patient-centric education and addressing individual patient needs (15). Current picture of the growing challenge of KSD, highlights the discrepancy between advancements in medical understanding and the real-world increase in disease prevalence and recurrence motivated present work.

Our study aims to bridge this gap by obtaining information on the requirements to improve patient care, both by the patients themselves and healthcare providers, and to review the literature on patient expectations in KSD management.

**MATERIEL AND METHODS**

**Study Design**

Our study employed a cross-sectional survey design to gather comprehensive data on the management of KSD. The aim was to capture a wide range of perspectives from both healthcare professionals and patients to understand the current practices and needs in KSD management.

**Survey Development**

The survey was collaboratively developed with input from experts in kidney stone disease, including nephrologists and urologists. The questionnaire comprised 10 carefully crafted questions designed to cover various aspects of KSD management, such as dietary habits, fluid intake, medication adherence, and lifestyle changes. The survey was developed using Microsoft software, ensuring a standardized and user-friendly format.

**Questions Format**

The questions were formulated to elicit responses based on the knowledge and experiences of the respondents. The multiple-choice questions were designed to gather specific information on practices and opinions. While the Likert scale questions gauged the level of agreement or importance participants attributed to various aspects of KSD management. We used the five -point scale (‘essential’, ‘very useful’, ‘useful’, ‘neutral’, ‘not useful’). This approach allowed us the quantification of subjective experiences, facilitating analysis and comparison of participants points of views on a topic.

Before distribution, the survey and its methodology were submitted to our local ethics committee and received approval from our board. We ensured the anonymization of the collected data to maintain participant confidentiality. The analysis was conducted with the primary purpose of enhancing the understanding of kidney stone management.

**Participant Recruitment and Data Collection**

The survey targeted a predefined group of healthcare professionals – nephrologists, urologists, biologists, and dietitians – who were contacted via their professional networks through email. Additionally, patients were interviewed to ensure they comprehended the questions during their regular consultation process. This approach facilitated gathering diverse perspectives from both practitioners and patients. The survey was open for responses from February to May 2022, spanning three months to allow adequate participation.

**Data Analysis and Presentation:**

All the responses recorded through the survey were analyzed using Microsoft software tools. The data were presented in a chart format, primarily using Excel software, for clear visualization and interpretation. Our analysis was primarily descriptive, focusing on frequencies and patterns in the responses.

**Utilization of Pareto Chart:**

A key component of our data presentation was the Pareto chart. This bar chart, sorted by frequency, included a line graph to represent cumulative scores. It is a tool commonly used in quality control settings to identify critical factors in a process. In our study, the Pareto chart was instrumental in identifying key factors in the management of kidney stones, providing valuable insights into areas requiring attention or improvement.

**RESULTS**

**Survey Participation and Demographics**

Through our survey and ancillary data collection methods, we received a total of 121 responses. After careful review, we identified and excluded one duplicate response from our dataset. Consequently, our final analysis was conducted with a robust sample of 120 participants. The professional distribution of the respondents was as follows: 45 nephrologists, 38 dietitians, 14 patients with kidney stones, 11 urologists, one biologist, and other specialties including one radiologist, one psychiatrist, two trade stakeholders, with the remaining being trainees in related fields (Figure 1A). The Pareto chart, a bar graph sorted by frequency with a cumulative line graph, is instrumental in identifying critical factors in a process—in this case, the management of kidney stones. It illustrates the distribution of healthcare provider specialties and the proportion of patient participants, highlighting the importance of nephrologists and dietitians. The chart also depicts the distribution of participants across various institutional categories, excluding patients. It underscores the significant role of academic, private, and public institutions in kidney stone management.

**Involvement in Kidney Stone Management**

Of the 86 professionals actively involved in kidney stone patient management, the majority (80.6%) see fewer than one patient per month. Approximately 6.3% see between 6 to 10 patients, 7.5% manage 11 to 25 patients, 10% handle 25 to 50 patients, and only three urologists reported managing more than 50 patients per month.

The primary reason cited by 60 participants for not being actively involved in kidney stone patient care was engagement in other types of medical activities. About 25% indicated a lack of experience in this area, and none of the respondents considered kidney stone disease management to be overly complicated or uninteresting.

**Adaptation of Water Intake and Patient** **Adherence**

A significant finding was the expressed need for adapting the daily volume of water intake for patients, which emerged as a primary consideration in patient care. This aspect, along with other specific questions related to improving kidney stone management and patient compliance, is elaborated in Figure 2.

**Key Findings from the Survey:**

**Daily Water Intake:**

The recommendation to adjust the daily water intake was overwhelmingly supported, with 97.5% of participants (including 93 respondents considering it 'essential,' 20 'very useful,' and 4 'useful') acknowledging its significance. Only two respondents were neutral, and one found it unhelpful.

It indicates a universal consensus on the critical role of hydration in KSD management.

**Dietary Advice:**

The importance of practical dietary advice was recognized by 94.1% of participants, with 83 considering it 'essential,' 26 'very useful,' and 4 'useful.' Neutral responses amounted to six, while only one participant found it unhelpful.

It is high recognition of its importance, but the data might suggest a gap in practical application or patient adherence.

**Reminders for Water Intake**

The idea of providing reminders, including the volume of water to drink and specific times for drinking, was regarded as valuable by 88.3% of participants (55 found it 'essential,' 38 'very useful,' and 13 'useful'). Fourteen participants were neutral, and none found it unhelpful.

Strong support for reminders, indicates the potential effectiveness of digital tools or interventions in improving hydration habits.

**Urine Color Monitoring**

Monitoring urine color was considered 'essential' by 30 participants, 'very helpful' by 57, and 'useful' by 15, comprising 85% of the total responses. Only 16 were neutral, and 2 considered it not useful.

Majority view it as a crucial part of self-management, suggesting that patients value simple, visual methods to monitor their condition.

**Urine pH Monitoring**

The utility of urine pH monitoring was acknowledged by 84.3% of respondents (27 found it 'essential,' 49 'very useful,' and 25 'useful'). There were 19 neutral responses and no negative responses.

Widely acknowledged as valuable, this data reflects awareness of the importance of biochemical monitoring in KSD.

**Urine Gravity Monitoring**

About 65.8% found monitoring urine gravity to be important, with 17 considering it 'essential,' 39 'very useful,' and 23 'useful.' Neutral and negative responses were 39 and 2, respectively.

Considered important, but with a notable number of neutral responses, suggesting a need for more education or clarity on its relevance.

**24-hour Urine Volume Monitoring**

This parameter was deemed important by 79.1% of participants (35 considered it 'essential,' 46 'very useful,' and 15 'useful'). Twenty-three were neutral, and only one found it unhelpful.

Recognized as important, highlighting the need for comprehensive monitoring in KSD management.

**Remote Monitoring of Urinary Parameters**

The concept of remote monitoring for urine color, pH, density, and 24-hour volume were viewed positively by 65% of participants. Neutral and negative views were held by 39 and 3 participants, respectively.

Positive views indicate an openness to technological solutions in managing KSD, though there is some hesitancy or lack of awareness about its potential.

**Fatigue Monitoring**

Monitoring fatigue was seen as 'essential' or 'very useful ' by 65% of patients, contrasting with the views of healthcare professionals (9% of urologists, 24% of nephrologists, and 52.6% of dietitians). Overall, 58.3% (10 'essential,' 34 'very useful,' and 26 'useful') supported this, with 41 neutral and 9 negative responses.

Varied responses, with patients valuing this more than healthcare professionals. This discrepancy points to a need for broader understanding of KSD's systemic effects.

**Mood Monitoring**

The majority (72.5%) considered mood monitoring 'essential' (15 participants), 'very useful ' (35), or 'useful ' (37). The response was 'neutral' for 29 and 'not useful at all' for 4. Again, a discrepancy was noted between patients (71% found it 'very useful' or 'essential') and healthcare professionals (18% of urologists, 28.8% of nephrologists, and 57.8% of dietitians).

Generally seen as important, particularly by patients, emphasizing the need to address the psychological aspects of KSD.

A significant finding was the divergence in perspectives between patients and healthcare professionals, particularly concerning fatigue and wellness monitoring. This highlights a gap in understanding patient experiences and the need for more patient-centric approaches in managing KSD.

**DISCUSSION**

Our comprehensive survey paints a multifaceted landscape of KSD management, highlighting the diverse yet often discordant perspectives of healthcare professionals and patients. This narrative not only illustrates the complexities inherent in KSD care but also underscores the pressing need for a more integrated, patient-centered approach.

*A Mosaic of Expert Perspectives*

Picture a scene where 45 nephrologists 38 dietitians, 11 urologists, and a smattering of other specialists, including patients themselves, converge. Each brings a unique thread to the tapestry of KSD management. The result is a rich, multi-dimensional picture of the disease, but one that also highlights the need for a more cohesive, interdisciplinary approach.

*In the Clinician’s Office*:

In this narrative, most healthcare professionals encounter kidney stone patients infrequently, with 80.6% seeing less than one patient per month. This rarity in their practice might contribute to an undercurrent of underestimation or even inadvertent neglect of KSD, hinting at a potential gap in routine healthcare. Delving deeper into the survey data reveals multifaceted and nuanced insights into KSD management, emphasizing the complexity of patient needs and the potential for improvements in care strategies.

*Hydration Management Nuances*

The near-universal recognition of the importance of water intake highlights a well-established medical consensus. However, the enthusiasm for reminders about water intake reveals an underlying challenge in patient behavior modification. This could suggest that despite understanding the importance of hydration, patients struggle with the practical implementation of these guidelines in their daily routines. The data might reflect a broader issue in chronic disease management - the gap between knowledge and consistent, long-term behavior change.

A study aimed at understanding kidney stone patients' experiences with increasing fluid intake, a well-established preventive strategy, reported that adherence to this recommendation is commonly below 50%. This indicates a significant gap in adherence to one of the most basic and cost-effective prevention strategies for kidney stones​​.

*Dietary Management Challenges*

The recognition of dietary advice's importance, contrasted with potential issues in adherence, points towards a disconnect between the provision of information and its practical application. This could be due to various factors, such as the complexity of dietary guidelines, socioeconomic barriers, or a lack of personalized dietary planning. However, 73.3% of patients adhered to the prescribed treatment (especially for hypocitraturia) and diet. The overall adherence rate was 61.2% for those treated with a single drug and 85.4% for those treated with multiple drugs. A significant increase in citrate levels was observed in patients with good adherence compared to non-adherent patients​​ (16). It suggests a need for more tailored dietary interventions, possibly involving more frequent and in-depth consultations with dietitians or the use of technology to provide personalized dietary recommendations.

*Comprehensive Urine Monitoring*

The emphasis on urine monitoring reflects an awareness of its importance in tracking treatment efficacy, disease progression and management. The varied responses across different urine parameters might indicate differing levels of patient understanding or perceived ease of monitoring these aspects. For example, urine color is a simpler parameter to track compared to pH or gravity, which might require more specific tools or kits. Urine and stone analysis are crucial for diagnosis and treatment, with 24-hour urine collection being the most useful method (17). This suggests an opportunity for educational interventions or the development of user-friendly home monitoring kits using the urine colour pe.

*Remote Monitoring and Digital Health*

Positive responses to remote monitoring align with global trends in telehealth and digital health adoption. This acceptance presents a significant opportunity for healthcare systems to invest in remote monitoring technologies, which could lead to more proactive and continuous management of KSD. However, the hesitancy or lack of awareness about its potential also suggests a need for patient education and demonstration of the efficacy of these tools. Despite the availability of numerous mobile applications for KSD, gaps in physician involvement, data security, and functionality persist. Effective advancement of mobile health tools for KSD requires oversight by urological associations and patient groups, ensuring regular updates in content and data security (18).

*Psychological Aspects in KSD*

The significant emphasis that patients place on fatigue and mood monitoring, compared to healthcare professionals, could indicate an under-addressed aspect of KSD management. This suggests that patients experience is a considerable psychological and emotional burden, which might be overlooked in routine clinical practice. Addressing these aspects could involve incorporating mental health screenings and support into KSD management protocols.

*Integrating Patient Perspectives*

The divergent views between healthcare professionals and patients underscore a critical gap in understanding and addressing patient experiences and expectations in KSD management. This indicates a need for more empathetic, patient-informed approaches in clinical practice, potentially involving patient advisory boards or more regular patient feedback mechanisms in care planning. Indeed, study focused on patients' perception of kidney stone prevention within the emergency department found that 68% of patients did not receive any instructions about prevention in the emergency department. However, among those who did receive instructions, adherence was higher among educated patients (90%), those with insurance coverage (85%), and those with an income higher than $1000 per month (76%)​​. A significant majority, 71%, have faith in the effectiveness of stone prevention measures when provided. Additionally, a larger proportion, 82%, express interest in learning more about these preventive strategies. However, the adherence to these measures is impeded by certain factors, with the primary reason being the cost, as indicated by 53.1% of the patients. This is followed by 18.8% of patients pointing out a lack of adequate explanation from emergency department physicians as another barrier. This data underscores the importance of patient education and cost-effective strategies in the management and prevention of kidney stones (19).

*Future Directions in KSD Management*

The data points towards a more holistic, patient-centered approach in KSD management, integrating medical, lifestyle, and psychological aspects. This could involve multidisciplinary teams including nephrologists, dietitians, mental health professionals, kidney stone nurses, and patient educators, working collaboratively to address the multifaceted needs of KSD patients.

Indeed patient-reported outcomes were prominently used in analgesic control studies, but less so in other areas in a recent systematic scoping review, covering literature from January 1, 2005, to March 30, 2021, aimed to evaluate the comparative effectiveness of various approaches in kidney stone disease (3). There is a clear call for innovation in patient education and engagement strategies, possibly through digital platforms that provide interactive, personalized content and support.

**Limitations:**

Our study acknowledges potential biases, including self-selection and non-response, limiting the generalizability of the findings.

**Conclusion:**

In essence, the survey data not only highlights the current challenges in KSD management but also opens avenues for innovative approaches that holistically address patient needs, integrating medical, dietary, technological, and psychological aspects into a cohesive management plan.

By integrating patient-centered strategies with continuous education and leveraging technological advancements, we can significantly enhance adherence and mitigate the impact of KSD.

**Disclosure statement:**

Dr. AP, has received a consultancy fee from Renal Care & Research sprl. This professional relationship has been conducted separately and has not influenced the data collection, analysis, or findings of this study.

**Funding and Acknowledgments**:

Preliminary findings of our study were showcased at the prestigious 22nd Symposium on Kidney Stones and Mineral Metabolism. This symposium, themed "Kidney Stone Disease in 2022: Bridging the Gap Between Knowledge and Practice," was held in Brussels, Belgium, in May 2022.

We thank the Brugmann Foundation for their financial support and acknowledge the contributions of all participants in the LUNNE meeting, especially Dr. Isabelle Tostivint, for their valuable insights.

**References:**

1. Thongprayoon C, Krambeck AE, Rule AD. Determining the true burden of kidney stone disease. Nat Rev Nephrol. 2020;16(12):736-46.

2. Pozdzik A, Maalouf N, Letavernier E, Brocheriou I, Body JJ, Vervaet B, et al. Meeting report of the "Symposium on kidney stones and mineral metabolism: calcium kidney stones in 2017". J Nephrol. 2019;32(5):681-98.

3. Dangle P, Tasian GE, Chu DI, Shannon R, Spiardi R, Xiang AH, et al. A Systematic Scoping Review of Comparative Effectiveness Studies in Kidney Stone Disease. Urology. 2023.

4. Lundeen C, Lim JRZ, Scotland KB, Ardekani RS, Penniston KL, Streeper NM, et al. What is the relationship of stress to patients' kidney stone-related quality of life? Can Urol Assoc J. 2021;15(5):E256-E60.

5. Spiardi R, Goldfarb DS, Tasian GE. Role of Climate Change in Urologic Health: Kidney Stone Disease. Eur Urol Focus. 2023;9(6):866-8.

6. Wang K, Ge J, Han W, Wang D, Zhao Y, Shen Y, et al. Risk factors for kidney stone disease recurrence: a comprehensive meta-analysis. BMC Urol. 2022;22(1):62.

7. Lotan Y. Economics and cost of care of stone disease. Adv Chronic Kidney Dis. 2009;16(1):5-10.

8. Roberson D, Sperling C, Shah A, Ziemba J. Economic Considerations in the Management of Nephrolithiasis. Curr Urol Rep. 2020;21(5):18.

9. Ghani KR, Rojanasarot S, Cutone B, Bhattacharyya SK, Krambeck AE. Economic burden of complicated ureteral stent removal in patients with kidney stone disease in the USA. J Comp Eff Res. 2022;11(17):1253-61.

10. Peerapen P, Thongboonkerd V. Kidney Stone Prevention. Adv Nutr. 2023;14(3):555-69.

11. Golomb D, Nevo A, Goldberg H, Ehrlich Y, Margel D, Lifshitz D. Long-Term Adherence to Medications in Secondary Prevention of Urinary Tract Stones. J Endourol. 2019;33(6):469-74.

12. Dauw CA, Yi Y, Bierlein MJ, Yan P, Alruwaily AF, Ghani KR, et al. Factors Associated With Preventive Pharmacological Therapy Adherence Among Patients With Kidney Stones. Urology. 2016;93:45-9.

13. Dauw CA, Yi Y, Bierlein MJ, Yan P, Alruwaily AF, Ghani KR, et al. Medication Nonadherence and Effectiveness of Preventive Pharmacological Therapy for Kidney Stones. J Urol. 2016;195(3):648-52.

14. Tasian GE, Kabarriti AE, Kalmus A, Furth SL. Kidney Stone Recurrence among Children and Adolescents. J Urol. 2017;197(1):246-52.

15. Langham RG, Kalantar-Zadeh K, Bonner A, Balducci A, Hsiao LL, Kumaraswami LA, et al. Kidney health for all: bridging the gap in kidney health education and literacy. Kidney Int. 2022;101(3):432-40.

16. Manzo BO, Cabrera JD, Emiliani E, Sanchez HM, Eisner BH, Torres JE. Impact of the adherence to medical treatment on the main urinary metabolic disorders in patients with kidney stones. Asian J Urol. 2021;8(3):275-9.

17. Williams JC, Jr., Gambaro G, Rodgers A, Asplin J, Bonny O, Costa-Bauza A, et al. Urine and stone analysis for the investigation of the renal stone former: a consensus conference. Urolithiasis. 2021;49(1):1-16.

18. Talyshinskii A, Bakhman G, Hameed BZ, Pietropaolo A, Naik N, Somani BK. Current state of mobile health apps in endourology: a review of mobile platforms in marketplaces and literature. Ther Adv Urol. 2023;15:17562872231176368.

19. Moussa M, Abou Chakra M. Patient's perception of kidney stone prevention within the emergency department and its adherence factors: a single institution study. BMC Emerg Med. 2019;19(1):48.
